# Supplementary material for: Impact of Somatic Vulnerability, Psychosocial Robustness and Injury-Related Factors on Fatigue following Traumatic Brain Injury—A Cross-Sectional Study
Source: J Clin Med. 2022 Mar 21;11(6):1733. doi: 10.3390/jcm11061733 (PMC8951420; doi:10.3390/jcm11061733)
Supplement: Supplementary file 1 [file jcm-11-01733-s001.zip › jcm-1634759-supplementary.pdf]

S1. Supplemental Materials

S1.1 Bivariate Associations between PROMS, Sociodemographic Variables, Injury Related factors, and Neuropsychological Measures

For the sake of brevity and clarity, bivariate associations between all included factors were not included in the main paper, but are supplied in the following section for those interested in the relations between the various fatigue PROMS and any particular variable. For the correlations between the fatigue scores on the various PROMS, see Table S1. The correlations between fatigue PROMS and sociodemographic and injury-related factors are supplied in Table S2. Correlations between fatigue PROMS and neuropsychological measures are supplied in Table S3. Finally, correlations between fatigue PROMS and PROMS of other related constructs are supplied in Table S4.

Table S1. Bivariate correlations between PROMS of fatigue. All correlations are significant at $p < .001$.

	FSS	CFQ Total	CFQ Physical	CFQ Mental	GSCL Fatigue
FSS-9	1				
CFQ Total	0.62	1			
CFQ Physical	0.63	0.96	1		
CFQ Mental	0.63	0.78	0.58	1	
GSCL Fatigue	0.63	0.77	0.76	0.55	1
RPQ Item 6	0.63	0.66	0.70	0.36	0.73

Table S2. Bivariate associations between fatigue PROMS, sociodemographic variables and injury-related factors (N = 96). ^{ns} = nearly significant, p < .08, * = p < .05

	FSS	CFQ Total	CFQ Physical	CFQ Mental	GSCL Fatigue	RPQ Item 6
Gender	0.15	0.10	0.16	-0.09	0.10	0.20 ^{ns}
Age (Centered)	-0.04	0.06	0.06	0.04	-0.09	0.04
Education (Centered)	0.00	-0.03	0.00	-0.09	-0.02	0.06
Discharge to Rehab (0/1)	0.07	0.24*	0.24*	0.17	0.05	0.06
AIS_head	0.13	0.20*	0.19^{ns}	0.15	0.11	0.12
Length of Acute Hospital Stay (days)	0.09	0.20*	0.23*	0.07	0.07	0.13
GCS at Discharge	-0.03	-0.16	-0.18^{ns}	-0.07	0.01	0.04
GOS at Discharge	0.13	-0.20^{ns}	-0.19 ^{ns}	-0.16	0.03	0.01
ASA_PS	0.06	0.10	0.10	0.08	0.01	0.09
Rotterdam CT-score	-0.03	0.06	0.11	-0.08	-0.04	0.01
Lowest GCS within 24 hours	-0.05	-0.10	-0.14	0.02	0.06	0.07
Multitrauma (0/1)	0.09	0.11	0.12	0.06	0.09	0.09

Table S3. Bivariate correlations between fatigue PROMS and neuropsychological measures. All neuropsychological measures are scaled scores according to the manual of each test, with the exception of a single calculated score from the CPT-III (CoV; Coefficient of Variation Block Change). Scaled T-scores from the CPT-III are reversed, so that higher scores equal better performance to align with polarity of other tests. N = 96 for most tests, with some exceptions (CWIT-1 = 91, CWIT-2 = 94, CWIT-3 & 4= 90, CPT-III = 95). ^{ns} = nearly significant, p < .08, * = p < .05, ** = p < .01

	FSS	CFQ Total	CFQ Physical	CFQ Mental	GSCL Fatigue	RPQ Item 6
WASI Matrix Reasoning	-0.04	0.01	0.05	-0.10	-0.04	0.11
WASI Similarities	-0.10	-0.09	-0.06	-0.14	-0.09	-0.03
WAIS-IV Digit Span Total	0.01	-0.12	-0.06	-0.21*	-0.06	-0.03
WAIS-IV Digit Span Forward	0.06	0.02	0.07	-0.11*	-0.06	0.02
WAIS-IV Digit Span Backward	-0.14	-0.13	-0.07	-0.22*	-0.07	0.02
WAIS-IV Digit Span Sequencing	0.01	-0.13	-0.11	-0.14	-0.08	-0.09
D-KEFS TMT-1	0.03	-0.11	-0.10	-0.11	-0.06	0.14
D-KEFS TMT-2	0.02	-0.08	-0.06	-0.09	0.02	0.10
D-KEFS TMT-3	-0.07	-0.09	-0.07	-0.11	-0.01	0.06
D-KEFS TMT-4 Time	-0.12	-0.12	-0.08	-0.18	-0.04	0.12
D-KEFS TMT-4 Errors	0.12	0.14	0.17	0.03	0.03	-0.01
D-KEFS TMT-5	-0.09	0.02	0.01	0.04	0.04	0.04
D-KEFS CWIT-1	-0.06	-0.16	-0.09	-0.27*	-0.03	0.06
D-KEFS CWIT-2	-0.10	-0.22	-0.17	-0.26*	-0.11	0.02
D-KEFS CWIT-3 Time	-0.11	-0.12	-0.10	-0.12	0.05	0.00
D-KEFS CWIT-3 Errors	-0.04	0.00	0.01	-0.03	0.06	0.02
D-KEFS CWIT-4 Time	-0.25*	-0.28**	-0.24*	-0.27**	-0.09	0.02
D-KEFS CWIT-4 Errors	-0.18	-0.14	-0.12	-0.13	-0.05	0.03
CPT-III Omissions	-0.17	-0.19^{ns}	-0.19	-0.12	-0.10	-0.13
CPT-III Commissions	-0.06	0.02	0.03	-0.01	0.02	0.03
CPT-III HRT	-0.07	-0.22*	-0.18	-0.23*	-0.02	-0.05
CPT-III HRT SD	-0.09	-0.23*	-0.18^{ns}	-0.28*	-0.02	-0.06
CPT-III Variability	-0.07	-0.16	-0.12	-0.21*	-0.01	-0.03
CPT-III HRT Block Change	0.00	-0.02	-0.04	0.03	0.02	0.07
CPT-III HRT ISI Change	0.16	-0.02	0.03	-0.14	0.09	-0.03
CPT-III CoV Block Change (raw score)	0.28**	0.18	0.15	0.19*	0.13	0.04

Table S4. Bivariate correlations between fatigue PROMS and PROMS of related constructs (N = 96). ^{ns} = nearly significant, p < .08, * = p < .05, ** = p < .01, *** = p < .001

	FSS	CFQ Total	CFQ Physical	CFQ Mental	GSCL Fatigue	RPQ Item 6
BAS Drive	-0,02	0,01	0,01	0,00	0,00	-0,05
BAS Fun Seeking	-0,04	0,01	-0,02	0,08	0,04	0,05
BAS Reward Responsiveness	-0,05	-0,05	-0,04	-0,05	-0,05	-0,10
BIS	0,26*	0,10	0,11	0,04	0,19^{ns}	0,13
ESQ	0,45***	0,32**	0,30**	0,28**	0,39***	0,32**
ISI	0,38***	0,46***	0,45***	0,34**	0,50***	0,36***
SCL Anxiety	0,41***	0,38***	0,37***	0,29**	0,49***	0,32***
SCL Depression	0,42***	0,43***	0,44***	0,27**	0,54***	0,46***
RSA Perception of Self	-0,33**	-0,22*	-0,23*	-0,11	-0,33**	-0,18^{ns}
RSA Planned Future	-0,39***	-0,35**	-0,34**	-0,25*	-0,36***	-0,26*
RSA Social Competence	-0,10	-0,04	-0,02	-0,06	-0,08	0,06
RSA Family Cohesion	-0,05	-0,08	-0,08	-0,06	-0,08	0,05
RSA Social Resources	-0,08	-0,03	-0,04	0,00	-0,14	0,07
RSA Structured Style	-0,20	-0,06	-0,06	-0,03	-0,12	-0,07
Pain – Affected Regions	0,39***	0,43***	0,46***	0,21*	0,52***	0,42***
NRS Strongest Pain	0,39***	0,32**	0,36***	0,13	0,55***	0,36***
NRS Weakest Pain	0,39***	0,29**	0,25*	0,30**	0,36***	0,34**
NRS Average Pain	0,42***	0,37***	0,35***	0,30**	0,58***	0,37***
NRS Current Pain	0,32**	0,35***	0,33**	0,29**	0,44***	0,36***
NEO Neuroticism	0,30**	0,20*	0,21*	0,11	0,31**	0,26*
NEO Extraversion	-0,13	-0,12	-0,12	-0,08	-0,22*	-0,07
NEO Openness	-0,02	0,08	0,09	0,05	0,06	0,25*
NEO Agreeableness	0,09	0,09	0,11	0,00	-0,02	0,03
NEO Conscientiousness	-0,18^{ns}	-0,05	-0,09	0,04	-0,09	-0,17
LOT-R Optimism	-0,19^{ns}	-0,16	-0,14	-0,14	-0,16	-0,03
UCLA Loneliness	0,22*	0,17	0,23*	-0,03	0,26**	0,14
GSCL Cardiovascular Symptoms	0,42***	0,44***	0,43***	0,32**	0,60***	0,43***
GSCL Gastrointestinal Symptoms	0,41***	0,28**	0,30**	0,16	0,54***	0,37***
GSCL Musculoskeletal Symptoms	0,55***	0,56***	0,55***	0,40***	0,74***	0,52***

S1.2 Primary Factor Loadings

For the primary factor analyses, the underlying dimensionality of all variables with significant loadings on one or more of the fatigue PROMS (outside of the CFQ Mental Fatigue subscale, as discussed in the main paper) was explored. Note that trait openness and the neuropsychological measures are not included, as they did not load saliently (above |0.40|) onto any factor. The factor loadings from the obliquely rotated factor structure is presented in Table S5.

Table S5. Structure matrix with variable loadings for the primay factor analysis after oblique rotation (Oblimin) (N = 96), with factor correlations. Variables with salient loadings on each factor (i.e. loadings above |0.40|) are marked in bold.

	Factors		
	Psychosocial Robustness	Somatic Vulnerability	Injury Severity
Behavioral Inhibition	**-0.55**	0.17	0.08
Trait Neuroticism	**-0.90**	0.29	0.06
Trait Extraversion	**0.64**	-0.13	-0.04
Trait Conscientiousness	**0.59**	-0.07	0.14
Trait Optimism	**0.67**	-0.12	-0.22
Loneliness	**-0.72**	0.17	0.03
Resilience – Perception of Self	**0.82**	-0.23	-0.13
Resilience – Planned Future	**0.63**	-0.38	-0.07
Anxiety Symptoms	**-0.64**	**0.52**	0.30
Depressive Symptoms	**-0.76**	**0.45**	0.26
Gastrointestinal Symptoms	**-0.51**	**0.61**	0.03
Cardiovascular Symptoms	**-0.53**	**0.53**	-0.01
Musculoskeletal Symptoms	-0.26	**0.84**	-0.02
Daytime Sleepiness	-0.19	**0.47**	-0.06
Insomnia Severity Index	-0.38	**0.48**	0.09
Pain – Affected Regions	-0.25	**0.73**	-0.11
Strongest Pain	-0.24	**0.84**	0.00
Weakest Pain	-0.09	**0.65**	0.11
Average Pain	-0.18	**0.88**	0.04
Current Pain	-0.10	**0.73**	-0.05
AIS_head	-0.02	0.06	**0.60**
Length of ICU Stay (days)	-0.16	0.06	**0.58**
GCS at Discharge	0.06	0.03	**-0.63**
GOSE at Discharge	-0.02	0.11	**-0.75**
Direct Pathway to Rehabilitation	-0.06	-0.07	**0.72**
**Factor Correlations**			
1. Psychosocial Robustness	1		
2. Somatic Vulnerability	-0.30	1	
3. Injury Severeity	-0.09	0.01	**1**

S1.3 Post-Hoc Regression Model

As outlined in the main manuscript, the residuals from the primary multiple regression of the fatigue factor correlated significantly with two neuropsychological measures (CWIT-4 and CPT-III CoV; Coefficient of Variation), meaning that these variables, while not loading onto any of the three factors from the factor analyses, are still associated with fatigue beyond the significant contributions of the factors. CWIT-4 is slightly more correlated with fatigue (N = 89, r = -0.27 p < 0.01) than CoV (N = 95, r = -0.20, p < 0.05), and CWIT-4 is also significantly correlated with CoV (N = 89, r = 0.28, p < 0.01). A post-hoc regression model was conducted to evaluate the additional explanatory value of neuropsychological function to the model. A composite score was created as the average of the summed standardized scores for CWIT-4 and CPT-III CoV, and entered in a final step in the regression model.

Table S6. Blockwise multiple regression (N = 89). Adjusted R^2 shows the model-explained variance, and the F Change-statistic is a test of the improvement from the previous model. Standard Errors (SE) shown are calculated from bootstrapping. The final column shows the 95% confidence interval for the unstandardized coefficients (B) in the model 4. ^{ns} = not significant, * = p < .05, ** = p < .01, *** = p < .001.

	**Model 1**		**Model 2**		**Model 3**		**Model 4**		95% CI	
	β	B (SE)	β	B (SE)	β	B (SE)	β	B (SE)	Lower	Upper
Constant		-.10 (.12)		-.10 (.12)		-.06 (.09)		-.07 (.09)	(-.23	.11)
Age (Centered)	.00	.00 (.01)	.00	.00 (.01)	-.04	-.00 (.01)	-.03	-.00 (.01)	(-.01	.01)
Education (Centered)	-.02	-.01 (.05)	-.01	-.00 (.05)	.10	.04 (.04)	.12	.05 (.04)	(-.02	.14)
Gender (Female)	.18	.43 (.24)	.17	.42 (.24)	.11	.27 (.18)	.10	.25 (.17)	(-.09	.57)
Injury Severity			.12	.14 (.11)	.18*	.20 (.08)	.13	.15 (.08)	(.00	.31)
Psychosocial Robustness					-.22*	-.23 (.08)	-.19*	-.20 (.08)	(-.35	-.04)
Somatic Vulnerability					.59***	.61 (.08)	.59***	.61 (.07)	(.48	76)
Neuropsychological Composite							-.22**	-.27 (.09)	(-.40	-.03)
Adjusted R^2	-.002		.002		.475		.516			
F Change	.93$^{ns.}$		1.34 $^{ns.}$		38.86***		7.94**			

S1.4 Post-Hoc Univariate Regression Analyses

While the factors derived from factor analyses do demonstrate significant associations with fatigue, these methods do not yield information about which of the underlying variables contribute to the association between the factor scores and fatigue. In the following section, several post hoc analyses are presented which were performed to illuminate and highlight those variables in each dimension most crucial to the dimensions' associations with fatigue. For the Psychosocial Robustness and Somatic Vulnerability factors, separate regression was performed including only one variable, and the resulting coefficients and percent of explained variance in the fatigue factor is presented in tables S7 and S8.

To examine the role of individual variables loading on the Injury Severity factor, the contribution of each variable was assessed as change in R^2 from a baseline model with age (centered), education (centered), gender (female), and the factor scores for Psychosocial Robustness and Somatic Vulnerability as baseline covariates. Additionally, the neuropsychological composite score from the previous section was analyzed alongside the individual neuropsychological scores. See table S9 for coefficients and R^2 change for all variables loading onto the Injury Severity factor and the neuropsychological composite score.

Table S7. Coefficients and explained variance in the fatigue factor (outcome variable) from univariate regression models with the Psychosocial Robustness factor and the individual variables loading onto this factor. N = 96 for all analyses.

	Univariate Regression Analyses		
	β	Adjusted R^2	Sig.
**Psychosocial Robustness**	**-.38**	**.14**	**< .001**
BIS	.17	.02	.090
Anxiety	.44	.19	< .001
Depression	.52	.26	< .001
RSA Perception of Self	-.31	.09	.002
RSA Planned Future	-.42	.17	< .001
NEO Neuroticism	.29	.08	.004
NEO Extraversion	-.16	.02	.116
NEO Conscientiousness	-.13	.01	.213
LOT-R Optimism	-.17	.02	.109
UCLA Loneliness	.24	.05	.017

Table S8. Coefficients and explained variance in the fatigue factor (outcome variable) from univariate regression models with the Somatic Vulnerability factor and the individual variables loading onto this factor. N = 96 for all analyses.

	Univariate Regression Analyses		
	β	Adjusted R^2	Sig.
**Somatic Vulnerability**	**0.63**	**0.39**	**< .001**
Daytime Sleepiness	.39	.14	< .001
Insomnia Severity	.46	.20	< .001
Pain – Affected Regions	.49	.23	< .001
NRS Strongest Pain	.48	.22	< .001
NRS Weakest Pain	.40	.15	< .001
NRS Average Pain	.48	.22	< .001
NRS Current Pain	.41	.16	< .001
GSCL Cardiovascular Symptoms	.50	.24	< .001
GSCL Gastrointestinal Symptoms	.45	.19	< .001
GSCL Musculoskeletal Symptoms	.66	.42	< .001

Table S9. Coefficients and explained variance in the fatigue factor (outcome variable) from univariate regression models with the Injury Severity factor and the individual variables loading onto this factor, as well as the neuropsychological measures and their composite. For these analyses, the variables were added to a baseline model with age, years of education, gender, Psychosocial Robustness and Somatic Vulnerability as covariates, and R^2 change reported is the change in explained variance from this baseline model.

	Univariate Regression Analyses				
	n	β	R^2 Change	Adjusted R^2	Sig.
**Injury Severity**	**96**	**.16**	**.03**	**.44**	**.041**
Direct Pathway to Rehabilitation	96	.18	.03	.45	.026
AIS_head	96	.17	.03	.44	.034
Length of Acute Hospital Stay (days)	96	.07	.00	.42	.420
GCS at Discharge	96	-.08	.01	.42	.308
GOSE at Discharge	96	-.11	.01	.43	.156
**Neuropsychological Composite**	**89**	**-.25**	**.06**	**.50**	**.002**
CWIT 4	90	-.23	.05	.49	.004
CPT-III CoV Block Change	95	-.16	.03	.45	.042
